# MicroRNA-298 Reverses Multidrug Resistance to Antiepileptic Drugs by Suppressing MDR1/P-gp Expression *in vitro*

**DOI:** 10.3389/fnins.2018.00602

**Published:** 2018-08-28

**Authors:** Yangmei Xie, Yiye Shao, Xiaolin Deng, Ming Wang, Yinghui Chen

**Affiliations:** ^1^Department of Neurology, Jinshan Hospital, Fudan University, Shanghai, China; ^2^Department of Neurology, Huashan Hospital North, Fudan University, Shanghai, China

**Keywords:** microRNA-298, P-glycoprotein, antiepileptic drugs, multidrug resistance, refractory epilepsy

## Abstract

P-glycoprotein (P-gp), a critical multidrug transporter, recognizes and transports various antiepileptic drugs (AEDs) through the blood-brain barrier (BBB). This may decrease the concentrations of AEDs in brain tissues and cause multidrug resistance (MDR) in patients with refractory epilepsy. Compelling evidence indicates that microRNAs (miRNAs) modulate MDR in various cancers by regulating P-gp expression. Furthermore, a previous study showed that miR-298 mediates MDR in breast cancer cells by downregulating P-gp expression. Based on the therapeutic results obtained from tumor cells, we aimed to determine whether miR-298 reverses MDR to AEDs by regulating P-gp expression in the BBB. We first established different drug-resistant cell lines, including PHT-resistant HBMECs (human brain microvascular endothelial cells) and doxorubicin (DOX)-resistant U87-MG (human malignant glioma) cells, by inducing P-gp overexpression. Quantitative real-time PCR (qRT-PCR) analysis revealed reduced expression of miR-298 in both HBMEC/PHT and U87-MG/DOX cells, and the luciferase reporter assay identified the direct binding of miR-298 to the 3′-untranslated region (3′-UTR) of P-gp. Moreover, ectopic expression of miR-298 downregulated P-gp expression at the mRNA and protein levels, thereby increasing the intracellular accumulation of AEDs in drug-resistant HBMEC/PHT and U87-MG/DOX cells. Thus, our findings suggest that miR-298 reverses MDR to AEDs by inhibiting P-gp expression, suggesting a potential target for overcoming MDR in refractory epilepsy.

## Introduction

Epilepsy is one of the most common neurological disorders, affecting approximately 50 million people worldwide (Henshall et al., 2016). Currently, epilepsy is mainly treated with antiepileptic drugs (AEDs). However, epidemiological data indicate that approximately 30% of patients with epilepsy exhibit a poor response to a variety of AEDs, although these drugs differ in their pharmacodynamics, pharmacological mechanisms and interaction potential. Patients exhibiting multidrug resistance (MDR) subsequently develop refractory epilepsy (Kwan and Brodie, 2000; Kwan et al., 2010) and suffer from severe psychosocial problems, intellectual dysfunction, a reduced quality of life, and even increased morbidity and mortality (French, 2007). Currently, the mechanisms underlying refractory epilepsy remain unclear. In the past few decades, several mechanisms of refractory epilepsy have been proposed, including the pharmacokinetic hypothesis, the neural network hypothesis, the seizure severity hypothesis, the gene variant hypothesis, and the transporter hypothesis (Tang et al., 2017). The transporter hypothesis is the most widely accepted and investigated theory (Potschka, 2012).

ATP-binding cassette (ABC) efflux transporters, which are expressed at high levels in endothelial cells in the BBB, maintain central homeostasis by limiting the delivery of substances from the blood to the brain. However, efflux transporters fail to distinguish between xenobiotic substances and therapeutic drugs, restricting the access of these drugs to the brain (Miller, 2010). ABC transporters are divided into three subfamilies, B, C, and G, among which P-glycoprotein (P-gp, ABCB1, or MDR1), multidrug resistance protein (MRP1, ABCC1), and breast cancer resistance protein (BCRP, ABCG2) are the most frequently investigated proteins that induce MDR in various diseases (Miller, 2015). In particular, the strongest evidence correlating transporter overexpression with resistance to AEDs has been obtained for P-gp. Based on accumulating evidence, P-gp is significantly overexpressed in brain tissue samples resected from patients and in models of refractory epilepsy (Brandt et al., 2006; Riganti et al., 2014). P-gp is a multi-specific efflux transporter that transports a variety of prescribed drugs. Several traditional AEDs, including phenobarbital (PB), phenytoin (PHT), lamotrigine (LTG), and levetiracetam (LEV), have been confirmed as substrates of P-gp, thereby restricting drug delivery to the brain and contributing to the development of refractory epilepsy (Luna-Tortos et al., 2008). The use of specific antagonists of P-gp or downregulation of P-gp expression has been shown to increase the concentrations of AEDs in the brain, thereby enhancing the therapeutic effects of these AEDs (Ma et al., 2013). However, the clinical use of exogenous drugs targeting P-gp is limited due to their side effects and lack of tissue specificity. Therefore, studies aiming to identify endogenous molecules that are capable of efficiently regulating P-gp expression to reverse MDR in patients with refractory epilepsy are urgently needed.

In recent years, microRNAs (miRNAs), a class of endogenous short non-coding RNAs of approximately 22–25 nt in length that regulate gene expression at the post-transcriptional level (Gisel et al., 2014), have received increasing attention. Notably, miRNAs modulate gene expression by imperfectly pairing with the 3′-untranslated region (3′-UTR) of their target mRNAs, leading to the degradation or inhibition of the translation of target genes (Bartel, 2009). According to a number of studies, miRNAs play a significant role in MDR by regulating P-gp expression in various diseases, particularly malignant tumors (Hong et al., 2013). However, few studies have focused on the role of miRNAs in reversing AED resistance in the central nervous system (CNS). In one study, miR-298 improved the response to doxorubicin (DOX) in human breast cancer by suppressing P-gp expression (Bao et al., 2012). Based on the effects of therapeutic strategies targeting P-gp on cancer, we set out to determine whether miR-298 reverses MDR to AEDs by regulating P-gp expression in subjects with refractory epilepsy. Efflux transporters in epileptogenic tissues were expressed not only by endothelial cells in the BBB but also by other brain parenchymal cells, such as astrocytes, microglia, and neurons (Liu et al., 2012). In the present study, we established drug-resistant human brain microvascular endothelial cells (HBMEC/PHT) and astrocytes (U87-MG/DOX), in which high expression of P-gp was induced, and used these cells to investigate the regulatory effects of miR-298 on P-gp expression and AED concentrations.

## Materials and Methods

### Cell Culture and Reagents

Human brain microvascular endothelial cells were purchased from ScienCell Research Laboratories (Carlsbad, CA, United States). Cells were grown in ECM medium (ScienCell, SC-1001, United States) supplemented with 5% fetal bovine serum, penicillin (100 U/ml)/streptomycin (100 μg/ml), and endothelial cell growth supplement (ECGS; 0.03 mg/ml) in a humidified 5% CO_2_ atmosphere at 37°C. HBMECs were continuously exposed to a high dose of PHT (20–40 μg/ml) for 2 weeks, as described in previous studies (Lu et al., 2004), to obtain the MDR phenotype. PHT was added to the HBMEC medium at 24 h after a normal passage. Cells underwent normal passaging after reaching 80% confluence within 5–6 days, and the PHT concentration was increased. The human malignant glioma cell line U87-MG was obtained from the cell bank of the Chinese Scientific Academy (Shanghai, China). U87-MG cells were cultured in Dulbecco’s Modified Eagle’s Medium (DMEM) supplemented with 10% fetal bovine serum (Invitrogen Corporation, Carlsbad, CA, United States). U87-MG cells were continuously exposed to stepwise increasing concentrations (5–100 ng/ml) of DOX for a period of 6 months. U87-MG cells were treated with DOX for 24 h, and then the medium was replaced with DOX-free culture medium and the culture was resumed for 3–5 days. After the cells grew to 90% confluence under stable growth, they underwent normal passaging, and the concentration of the DOX was increased. The above procedures were repeated until the cell clones were able to tolerate 100 ng/ml or higher concentrations of DOX. Finally, the drug-resistant HBMEC/PHT and U87-MG/DOX cells were selected and verified using the CCK-8 assay. DOX was purchased from Sigma Chemical Co. (St. Louis, MO, United States). PB was obtained from Shanghai New Asia Pharmaceutical Company, and PHT, LEV, and LTG were purchased from Meilunbio (Dalian Meilun Biotechnology).

### Cell Viability Assay

Cell viability was measured using a cell counting kit-8 assay (Dojindo, Japan). HCMEC and HCMEC/PHT cells were seeded in a 96-well plate at a density of 8 × 10^3^ cells/well and allowed to adhere overnight at 37°C. The culture medium was then replaced and treated with six different concentrations (0.01, 0.1, 0.5, 1, 5, and 10 mg/ml of phenytoin. U87-MG and U87-MG/DOX cells were seeded in a 96-well plate at a density of 3 × 10^3^ cells/well and allowed to adhere overnight at 37°C. The culture medium was then replaced and treated with five different concentrations (0.1, 1, 10, 100, and 1000 ng/ml) of DOX. After 48 h of incubation, cells in each well were stained with 10 μL of the CCK-8 solution diluted in 90 μL of growth medium. Then, cells were incubated at 37°C for 2 h, and the absorbance was measured at 450 nm using a plate reader (BioTek Epoch, Winooski, VT, United States). Each experiment was repeated at least three times.

### Transient Transfection of Cells

The miR-298 mimic and negative control miRNA (miR-NC, the scrambled sequence of the miR-298 mimic) were purchased from RiboBio (Guangzhou, China). HCMEC/PHT and U87-MG/DOX cells were seeded into 6-well plates and allowed to adhere for 24 h. Cells were then transfected with 10 nM miR-298 mimic or 10 nM miR-NC using Lipofectamine iMax (Invitrogen, Carlsbad, CA, United States), according to the manufacturer’s instructions. The medium was replaced with growth medium 6 h after transfection, and the cells were harvested 48 h after transfection for subsequent mRNA and protein expression analyses.

### Immunofluorescence Staining

For immunofluorescence (IF) staining, cells were seeded on cover slips in 24-well plates overnight, then fixed with 4% paraformaldehyde in phosphate-buffered saline (PBS) for 10 min, washed twice with PBS, and permeabilized with 0.1% Triton X-100 in PBS for 10 min. Fixed cells were pre-incubated with PBS containing 5% BSA for 40 min at room temperature. Cells were incubated with a primary antibody (anti-P-gp monoclonal antibody, 1:200 dilution; Santa Cruz Biotechnology) overnight at 4°C. Cells were then stained with a FITC-conjugated secondary antibody for 1 h at 37°C. DAPI (0.1 μg/ml) was added to the secondary antibody mixture to visualize nuclei. Fluorescence images were collected and analyzed using a confocal laser scanning microscope (Leica, Germany).

### Quantitative Real-Time PCR Analysis

Total RNA was isolated from the cells using TRIZOL Reagent (Takara, Japan) according to the manufacturer’s protocol. Next, the miR-298 cDNA was synthesized using a Mir-X miRNA First-Strand Synthesis Kit (Takara, Japan), and the cDNAs corresponding to the target genes ABCB1, ABCG2, and ABCC1 were synthesized using PrimeScript^TM^ RT Master Mix (Takara, Japan). Subsequently, miR-298 expression was detected using the Mir-X miRNA qRT-PCR SYBR Kit (Takara, Japan) in an Applied Biosystems 7300 instrument (Applied Biosystems, Foster City, CA, United States), with U6 snRNA as an internal quantitative control. The expression of target mRNA was measured using SYBR Premix Ex Taq (Tli RNaseH Plus; Takara), with β-actin as an internal control. The primers used in the study are shown in **Table 1**.

**Table 1:** The sequences of primers used in the current study.

Gene	Primer sequence (5′-3′)
ABCB1	F:5′-TTTTCATGCTATAATGCGAC-3′
	R:5′-TCCAAGAACAGGACTGATGG-3′
ABCG2	F:5′-CTTGGCTGAGGGTTTGGAACT-3′
	R:5′-GAATGTTCCAGAAATGGTGCAAGA-3′
ABCC1	F:5′-TTTATAGGATGAAATGAGGGTATAGT-3′
	R:5′-AACAACCCAACCAACCACCTCT-3′
ACTIN	F:5′-CATTGCCGACAGGATGCAG-3′
	R:5′-CTCGTCATACTCCTGCTTGCTG-3′
miR-298	F:5′-AGCAGAAGCAGGGAGGUUCUCCCA-3′
U6	F:5′-CTCGCTTCGGCAGCACA-3′
	R:5′-AACGCTTCACGAATTTGCGT-3′
MDR1 3′-UTR	F:5′-GCGCCCGCGATCGCGAATTCACTCTGACTGTATGAGATGT-3′
	R:5′-CTGCTCGAACTAGTCTCGAGCCAGTCACATGAAAGTTTAG-3′


### Western Blot Analysis

Total proteins were extracted from cultured cells using SDS lysis buffer (Beyotime, Shanghai, China). Equal amounts of protein were analyzed by 8% SDS-polyacrylamide gel electrophoresis and transferred to polyvinylidene difluoride membranes. After blocking with 5% non-fat milk at room temperature for 1 h, membranes were incubated with the following primary antibodies overnight at 4°C: anti-P-gp (1:1000; Cell Signaling Technology, United States), anti-MRP1 (1:500; Abcam, United States), anti-BCRP (1:500; Millipore, United States), anti-α-tubulin, and anti-β-actin (1:5000; Proteintech Group, United States). The appropriate peroxidase-conjugated antibodies (anti-mouse or anti-rabbit) were then incubated with the membranes at room temperature for 2 h. Reactive bands were detected using ECL-Plus reagent (Merck Millipore, Darmstadt, Germany), and the band intensity was quantified using a Bio-Rad 2000 gel imaging system equipped with QUANTITY ONE software (Bio-Rad Laboratories, Hercules, CA, United States).

### Luciferase Reporter Assay

The full-length human MDR1 3′-UTR containing the predicted miR-298 binding site (445–451 bp) was amplified from the genomic DNA using specific primers (**Table 1**). The Duo-Luciferase reporter vector pEZX-FR2 (GeneCopoeia Inc., Rockville, MD, United States) was linearized with *Eco*RI and *Xho*I restriction enzymes, and the PCR product was cloned into the linearized vector using an EasyGeno Assembly Cloning kit (Tiangen Biotech Co., Ltd., Beijing, China) to create the vector pEZX-MDR1-3′-UTR. The consensus miR-298 binding site was mutated using a QuikChange II site-directed mutagenesis kit (Stratagene, Agilent Technologies, Santa Clara, CA, United States) to construct a clone of the mutant MDR1 3′-UTR, which was referred to as pEZX-MDR1-3′-UTR-mut. All clones were verified by DNA sequencing. For the luciferase reporter assay, HEK293T cells seeded in 24-well plates were transiently transfected with pEZX-MDR1-3′-UTR or pEZX-MDR1-3′-UTR-mut reporter plasmids together with a miR-298 mimic or miR-NC using Lipofectamine iMax (Invitrogen, Carlsbad, CA, United States). Twenty-four hours after transfection, cells were lysed, and their luciferase activities were determined using a Luc-Pair^TM^ Duo-Luciferase Assay Kit (GeneCopoeia), according to the manufacturer’s instructions.

### Determination of Intracellular AED Accumulation Using HPLC

HCMEC/PHT and U87-MG/DOX cells were seeded in 6-well plates (3 × 10^5^ and 1 × 10^5^ cells/well, respectively) and allowed to adhere for 24 h. Cells were then transfected with 10 nM miR-298 mimic or 10 nM miRNA negative control using Lipofectamine iMax (Invitrogen, Carlsbad, CA, United States). The medium was changed to growth medium containing different AEDs 6 h after transfection. Then, the cell deposits were harvested 48 h after transfection and washed twice with PBS. The samples were mixed with 50 μl of methanol and centrifuged at 15,000 rpm for 10 min at 4°C to obtain the supernatant for LC-MS-MS analysis. The injection volume was 20 μl and a Waters BEH-C18 (2.1 mm × 50 mm × 1.7 μm) column was used for the analysis at 45°C. Chromatographic separation was conducted using the mobile phase consisting of water with 0.1% formic acid and acetonitrile at a flow rate of 0.4 ml/min. The concentrations of AEDs in the supernatant were determined by comparison with a standard curve derived from a standard solution of AEDs using an Agilent LC system (Palo Alto, CA, United States).

### Statistical Analysis

Statistical analyses were performed using SPSS 17.0 (SSPS, Inc., Chicago, IL, United States). A two-sided unpaired Student’s *t*-test was used to compare the differences between two groups and one-way ANOVA was used to compare the differences between multiple groups. Values are means ± SEM, and statistical significance was defined as *p* < 0.05 for all tests.

## Results

### Elevated P-gp Expression in Drug-Resistant HCMEC/PHT and U87/MG Cells

**FIGURE 1:**
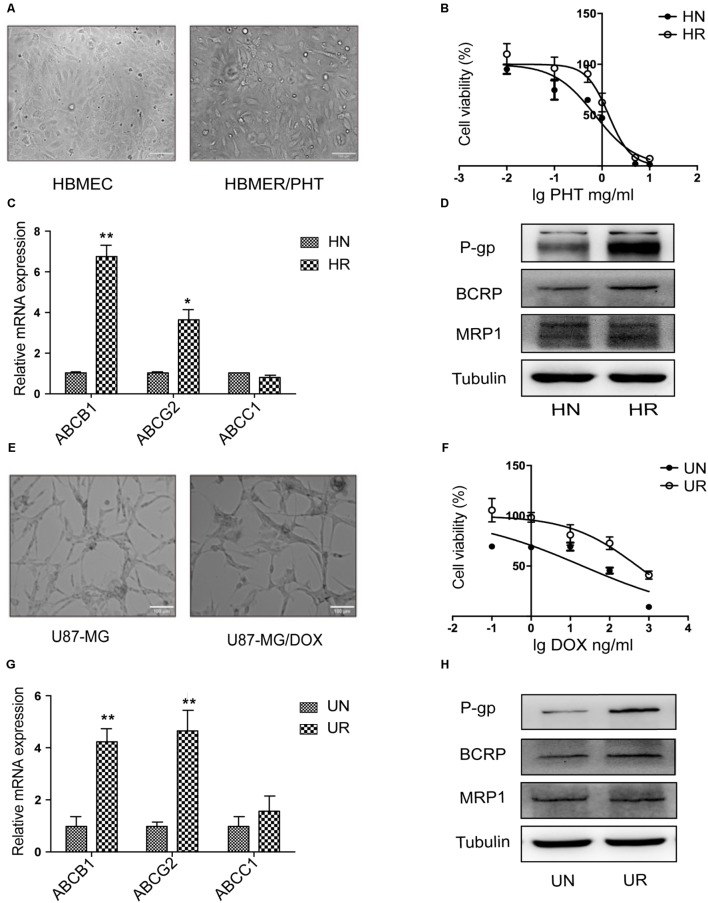
MDR phenotype of HBMEC/PHT and U87-MG/DOX cells. **(A)** Representative images showing the morphology of HBMEC, HBMEC/PHT, **(E)** U87-MG and U87-MG/DOX cells. **(B)** Viability of HBMEC, HBMEC/PHT cells, **(F)** U87-MG and U87-MG/DOX cells, as measured by the CCK-8 assay. **(C)** Levels of the ABCB1, ABCG2, and ABCC1 mRNAs in HBMEC (HN), HBMEC/PHT (HR). **(G)** U87-MG (UN), and U87-MG/DOX (UR) cells, as measured by qRT-PCR. **(D)** Representative western blots showing the levels of the P-gp, BCRP, and MRP1 proteins in HBMEC, HBMEC/PHT, **(H)** U87-MG, and U87-MG/DOX cells. (^∗^*p* < 0.05 and ^∗∗^*p* < 0.01 unpaired Student’s *t*-tests).

According to convincing data, overexpression of ABC efflux transporters in the BBB contributes to MDR in various CNS diseases (Miller, 2015). In the present study, a marked enlargement of the cell body was observed in drug-resistant HBMEC/PHT (**Figure 1A**) and U87-MG/DOX cells (**Figure 1E**). We first analyzed the cytotoxicity of PHT or DOX toward the two different cell lines using the CCK-8 assay to determine the MDR phenotype of HCMEC/PHT and U87-MG/DOX cells. As shown in **Figures 1B,F** higher viability was observed for HCMEC/PHT and U87-MG/DOX cells than the parental cells, and the resistance indices of the two cell lines were 1.95 and 25, respectively. Next, we detected the expression of MDR mRNAs and proteins, including P-gp, BCRP, and MRP1, in the two drug-resistant cell lines using qRT-PCR and western blotting, respectively. The drug-resistant cells exhibited markedly increased expression of the P-gp and BCRP mRNAs and proteins compared with their parental counterparts, whereas MRP1 expression was not significantly different (**Figures 1C,D,G,H**). Based on these data, the drug resistance of HCMEC/PHT and U87-MG/DOX cells may be attributed to elevated P-gp and BCRP expression.

### MiR-298 Inhibits P-gp Expression by Directly Targeting the 3′-UTR of P-gp

**FIGURE 2:**
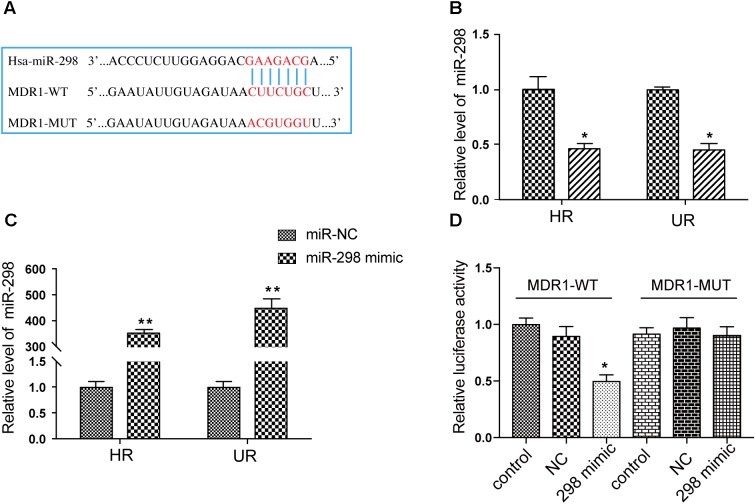
Validation of the MDR1 3′-UTR as a direct target of miR-298. **(A)** The predicted miR-298 binding site within the 3′-UTR of MDR1. **(B)** The expression of miR-298 in HBMEC/PHT and U87-MG/DOX cells compared with their sensitive counterparts, as measured by qRT-PCR. **(C)** The miR-298 level in HBMEC/PHT and U87-MG/DOX cells after miR-298 transfection compared with miR-NC, as measured by qRT-PCR. **(D)** Luciferase activity was analyzed as the ratio of relative Firefly to Renilla luciferase activities following co-transfection of the wildtype or mutant MDR1-3′-UTR construct. (^∗^*p* < 0.05, ^∗∗^*p* < 0.01 unpaired Student’s *t*-tests and one-way ANOVA).

As shown in recent studies, miRNAs modulate MDR in multiple diseases by regulating P-gp expression (Hong et al., 2013). The miR-298 level was markedly reduced in drug-resistant HCMEC/PHT and U87-MG/DOX cells compared with the respective control cell populations (**Figure 2B**), which was inversely correlated with P-gp expression. Thus, miR-298 may be a suppressor of P-gp. We predicted the potential binding site of miR-298 within the 3′-UTR of MDR1 using online databases to corroborate the miR-298-mediated modulation of P-gp expression (**Figure 2A**). Next, the full-length MDR1 3′-UTR was cloned from HCMEC/PHT cells to generate a MDR1-3′-UTR luciferase reporter, which was then co-transfected with the miR-298 mimic or miR-NC in HEK293T cells. The activity of the MDR1-3′-UTR luciferase reporter was decreased by approximately 50% in HEK293T cells transfected with the miR-298 mimic compared with miR-NC-transfected cells. However, the luciferase activity of cells transfected with the miR-298 mimic and pEZX-MDR1-3′UTR-mut was not significantly different from miR-NC-transfected cells (**Figure 2D**). Therefore, the 3′-UTR of MDR1 is a direct target of miR-298.

### Overexpression of miR-298 Downregulates P-gp Expression in HCMEC/PHT and U87/DOX Cells

**FIGURE 3:**
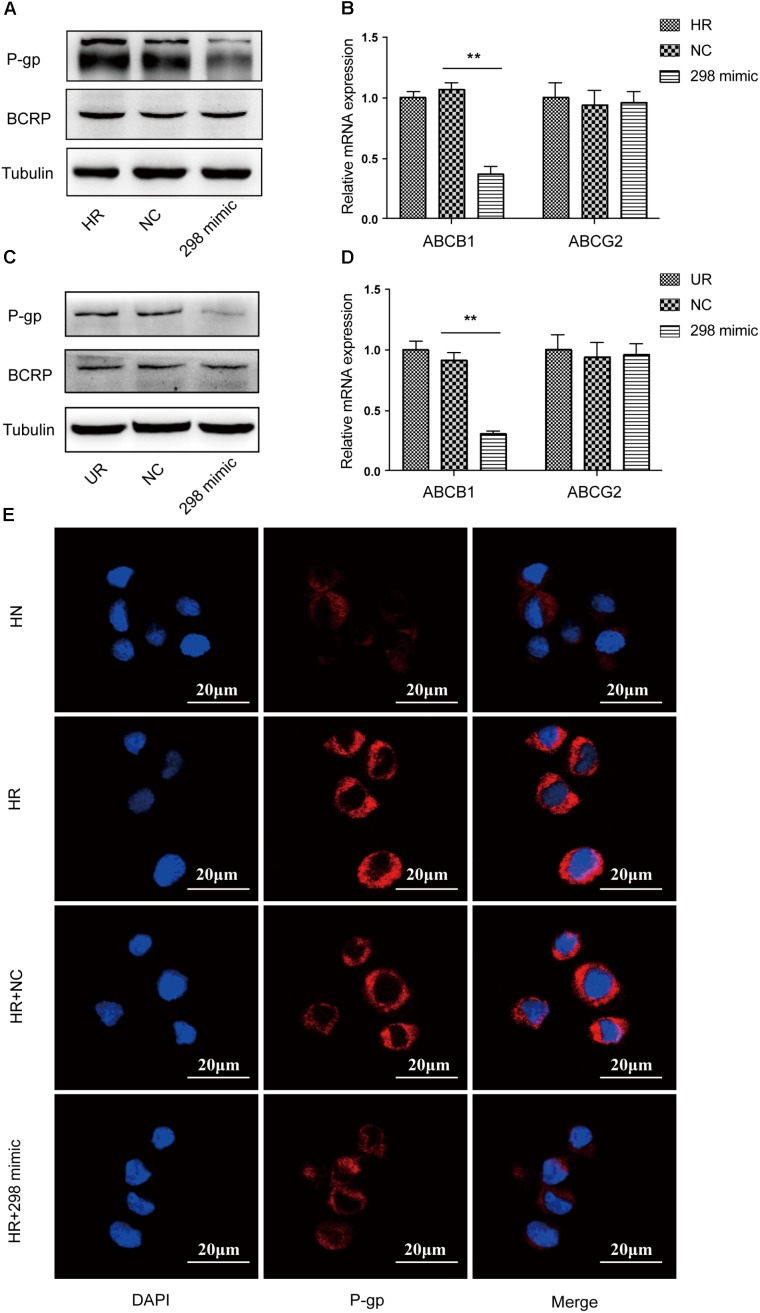
Effect of miR-298 on the regulation of MDR1/P-gp expression. **(A)** Representative western blot and **(B)** qRT-PCR analysis showing the expression of P-gp and BCRP after miR-298 transfection in HBMEC/PHT cells. **(C)** Representative western blot and **(D)** qRT-PCR analysis showing the expression of P-gp and BCRP after miR-298 transfection in U87-MG/DOX cells. **(E)** Representative image of P-gp expression after miR-298 transfection in HBMEC/PHT cells by IF. (^∗^*p* < 0.05 and ^∗∗^*p* < 0.01 unpaired Student’s *t*-tests and one-way ANOVA).

We first transfected the miR-298 mimic or miR-NC into HCMEC/PHT and U87-MG/DOX cells to confirm whether miR-298 regulates P-gp/MDR1 expression. Notably, miR-298 expression was increased more than 320-fold at 48 h after transfection with the miR-298 mimic compared with miR-NC (**Figure 2C**). The expression of the P-gp mRNA and protein was markedly decreased in cells transfected with the miR-298 mimic compared with the cells transfected with miR-NC (**Figures 3A–D**). However, BCRP expression was not significantly altered. According to the immunofluorescence staining, miR-298 also inhibited P-gp expression in HCMEC/PHT cells (**Figure 3E**). Based on these results, miR-298 downregulates P-gp expression at the mRNA and protein levels.

### Ectopic miR-298 Expression Increases the Concentrations of AEDs in HCMEC/PHT and U87/DOX Cells

**FIGURE 4:**
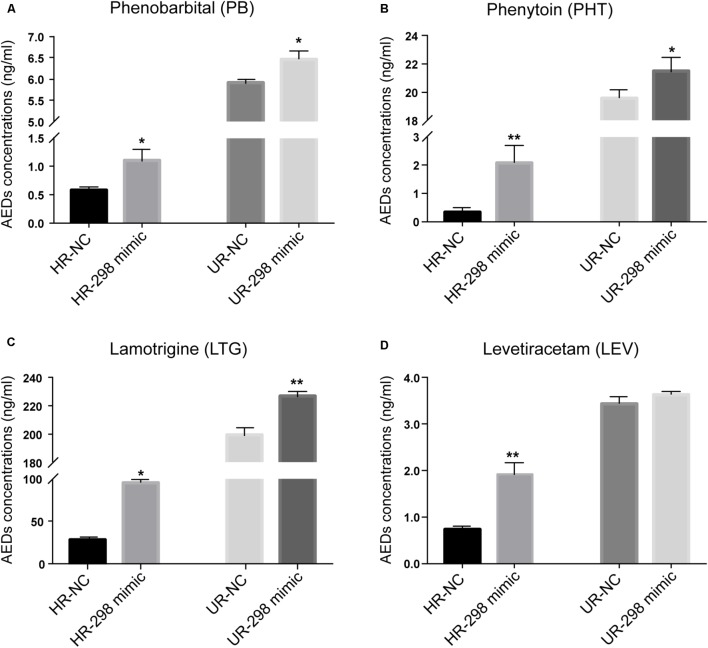
Regulatory effect of miR-298 on AED concentrations in HCMEC/PHT and U87-MG/DOX cells. **(A)** Phenobarbital (PB), **(B)** Phenytoin (PHT), **(C)** Lamotrigine (LTG), and **(D)** Levetiracetam (LEV). (^∗^*p* < 0.05, ^∗∗^*p* < 0.01 unpaired Student’s *t*-tests and one-way ANOVA).

Multidrug resistance in epilepsy was hypothesized to be partially attributed to P-gp overexpression, restricting the access of AEDs to the brain (Potschka and Luna-Munguia, 2014). Different concentrations of AEDs, including PB, PHT, LTG, and LEV, were added to the cultures of drug-resistant cells transfected with the miR-298 mimic to determine whether miR-298 reversed the MDR to AEDs by inhibiting P-gp expression. Then, the intracellular concentrations of the four AEDs in HCMEC/PHT and U87/DOX cells were examined. Ectopic miR-298 expression markedly increased the accumulation of PB, PHT, and LTG in the two types of drug-resistant cells (**Figures 4A–C**). However, overexpression of miR-298 increased the concentration of LEV in HCMEC/PHT cells, but not in U87/DOX cells (**Figure 4D**). Thus, miR-298 is a potential target for the reversal of MDR to AEDs *in vitro*.

## Discussion

The management of refractory epilepsy remains an urgent medical challenge that burdens our entire society. However, the mechanism of refractory epilepsy might be complex and multifactorial and remains to be elucidated. Recently, the transporter hypothesis has been investigated in several studies. According to several *in vitro* and *in vivo* investigations, complex mechanisms involving genetic factors, oxidative stress, inflammation, glutamate, and ligand-activated nuclear receptors underlie the upregulation of transporters such as P-gp in the BBB (Aronica et al., 2012). Additionally, repeated exposure to various drugs, such as chemotherapeutic agents and AEDs, induces the overexpression of multidrug transporters, which is widely used to establish multidrug-resistant cell models. In the present study, the expression of P-gp and BCRP was significantly elevated in drug-resistant cells. However, resistance to AEDs was not induced by BCRP overexpression in endothelial cells *in vitro* (Cerveny et al., 2006). In fact, data describing the induction of P-gp expression by AEDs *in vitro* remains controversial. Exposure to major AEDs, such as phenobarbital, phenytoin, and carbamazepine, did not alter P-gp function in some studies. These inconsistencies may be explained by the use of different models, methodologies and analytical methods in different studies. The anticonvulsant effects of PHT and PB were recently shown to be significantly improved by the co-administration of a selective inhibitor of P-gp to rats with chronic intractable epilepsy (Brandt et al., 2006; van Vliet et al., 2006). Additionally, as shown in a few clinical reports, verapamil, a known P-gp inhibitor, reduces the seizure frequency in patients with drug-resistant epilepsy as an adjunct treatment (Iannetti et al., 2005; Nicita et al., 2014, 2016). Nevertheless, P-gp inhibitors eventually failed to be translated into clinical practice as a treatment for refractory epilepsy due to their obvious side effects. According to several recent studies, miRNAs modulate MDR by altering the expression of ABC transporters, such as BCRP and P-gp, in tumor cells (Haenisch et al., 2014). For example, miR-129 and miR-145 inhibit P-gp expression and sensitize human cancer cells to chemotherapy drugs by directly targeting the 3′-UTR of MDR1 (Ikemura et al., 2013; Lu et al., 2017). Additionally, the overexpression of miR-506 and miR-138 have been reported to indirectly downregulate P-gp expression by regulating different signaling pathways (Requenez-Contreras et al., 2017; Zhou et al., 2017). In a previous report, miR-298 ameliorated the chemoresistance of breast cancer cells by downregulating P-gp expression, which was markedly decreased in both HBMEC/PHT and U87-MG/DOX cells in the present study (**Figure 2B**). Nevertheless, further studies are needed to clarify whether miR-298 reverses resistance to AEDs in patients with epilepsy by regulating P-gp expression due to complications arising from the tissue-specific regulation of miRNAs; the same miRNA may have different functions in different cell types (Landgraf et al., 2007). Furthermore, the length of the MDR1 3′-UTR, a critical regulatory site for miRNA interactions, is highly variable among different cell lines. A shorter MDR1 3′-UTR has been observed in drug-resistant cells compared with the corresponding sensitive cell populations, which might induce loss of miRNA-dependent translational inhibition and lead to increased P-gp expression (Bruhn et al., 2016). Therefore, we amplified full-length human MDR1 3′-UTR from the genomic DNA of PHT-resistant HBMECs to construct a luciferase reporter for MDR1. The overexpression of miR-298 significantly inhibited MDR1-3′-UTR-luciferase reporter activity, while no obvious difference was observed in the mutated counterparts, indicating that miR-298 directly bound the 3′-UTR of P-gp (**Figure 3D**). Consistent with the results from a previous study, miR-298 dramatically suppressed P-gp expression at the mRNA and protein levels. Based on accumulating evidence, P-gp plays a critical role in refractory epilepsy because most of the commonly used AEDs are substrates of P-gp, resulting in therapeutic failure in patients with epilepsy by limiting drug delivery to epileptogenic brain regions. Convincing *in vivo* and *in vitro* data have confirmed that PB, PHT, and LTG are transported by P-gp (Zhang et al., 2012). LEV was found to be a substrate in human models, but not in animal models, using a modified version of the Transwell assay (Luna-Tortos et al., 2008). Consistent with previous studies, miR-298 overexpression significantly increased PB, PHT, and LTG concentrations in drug-resistant cells. Interestingly, LEV concentrations increased in HCMEC/PHT cells, but not in U87-MG/DOX cells, compared with their sensitive counterparts. This discrepancy might be explained by the fact that LEV is not a high-affinity substrate of P-gp. Furthermore, P-gp is expressed at far lower levels in astrocytes than in endothelial cells.

Our study is not without limitations. We chose the human malignant glioma cell line U87-MG to establish a drug-resistant cell model due to the poor availability and limited passaging capability of primary human astrocytes. In addition, one miRNA may have multiple mRNA targets; therefore, miR-298 may participate in diverse pathological processes, increasing the potential for undesired side effects of miRNA-based therapy. In contrast, the 3′-UTR of MDR1 is recognized by multiple miRNAs, and thus miRNA-298 is likely only one of several regulatory miRNAs (Filipowicz et al., 2008) and may not be sufficient to affect AED efficacy *in vivo* due to its weak regulation. Thus, further *in vivo* experiments are needed to completely characterize the role of miR-298.

**FIGURE 5:**
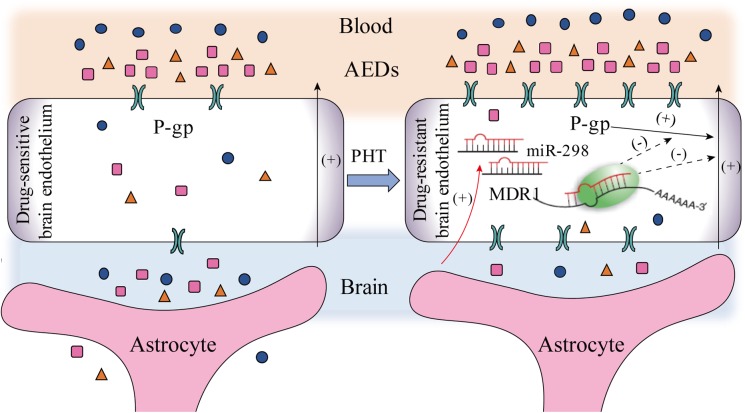
The potential mechanisms by which miR-298 reverses multidrug resistance to AEDs by regulating P-gp expression in patients with refractory epilepsy (activation steps are presented as solid lines and inhibitory effects are presented as dashed lines).

In summary, miR-298 increases the concentrations of AEDs in drug-resistant cells by directly inhibiting P-gp expression. As shown in **Figure 5**, miR-298 is a potential target to reverse MDR to AEDs *in vitro*.

## Author Contributions

YX performed most of the experiments and wrote the manuscript. YS and XD participated in the statistical analysis. MW provided important intellectual contributions and revised the manuscript. YC conceived the study and supervised the experiments. All authors have read and approved the final manuscript.

## Conflict of Interest Statement

The authors declare that the research was conducted in the absence of any commercial or financial relationships that could be construed as a potential conflict of interest.
